# Head CT Guidelines Following Concussion among the Youngest Trauma Patients: Can We Limit Radiation Exposure Following Traumatic Brain Injury?

**Published:** 2018-05-18

**Authors:** Bryan J. Harvell, Stephen D. Helmer, Jeanette G. Ward, Elizabeth Ablah, Raymond Grundmeyer, James M. Haan

**Affiliations:** 1 University of Kansas School of Medicine-Wichita, Department of Surgery; 2 Via Christi Hospital Saint Francis, Wichita, KS; 3 Chandler Regional Medical Center, Chandler, AZ; 4 University of Kansas School of Medicine-Wichita, Preventive Medicine and Public Health

**Keywords:** trauma, brain concussion, x-ray computed tomography, radiation, pediatrics

## Abstract

**Introduction:**

Recent studies have provided guidelines on the use of head computed tomography (CT) scans in pediatric trauma patients. The purpose of this study was to identify the prevalence of these guidelines among concussed pediatric patients.

**Methods:**

A retrospective review was conducted of patients four years or younger with a concussion from blunt trauma. Demographics, head injury characteristics, clinical indicators for head CT scan (severe mechanism, physical exam findings of basilar skull fracture, non-frontal scalp hematoma, Glasgow Coma Scale score, loss of consciousness, neurologic deficit, altered mental status, vomiting, headache, amnesia, irritability, behavioral changes, seizures, lethargy), CT results, and hospital course were collected.

**Results:**

One-hundred thirty-three patients (78.2%) received a head CT scan, 7 (5.3%) of which demonstrated fractures and/or bleeds. All patients with skull fractures and/or bleeds had at least one clinical indicator present on arrival. Clinical indicators that were observed more commonly in patients with positive CT findings than in those with negative CT findings included severe mechanism (100% vs. 54.8%, respectively, p = 0.020) and signs of a basilar skull fracture (28.6% vs. 0.8%, respectively, p = 0.007). Severe mechanism alone was found to be sensitive, but not specific, whereas signs of a basilar skull fracture, headache, behavioral changes, and vomiting were specific, but not sensitive. No neurosurgical procedures were necessary, and there were no deaths.

**Conclusion:**

Clinical indicators were present in patients with positive and negative CT findings. However, severe mechanism of injury and signs of basilar skull fracture were more common for patients with positive CT findings.

## Introduction

Annually, nearly 1.5 million children in the United States aged14 years and younger sustain a traumatic brain injury.^1^ A mild traumatic brain injury (MTBI) is defined as a complex pathophysiologic process induced by traumatic biomechanical forces secondary to direct or indirect forces.^2^ Rates of MTBI are highest for children aged four years and under.^1^ This age group is a difficult population to examine due to limited verbal skills placing them at particular risk for a missed diagnosis.^1^ Most traumatic brain injuries sustained by children four years and younger are minor and not associated with intracranial brain injury.^3^–^7^ However, MTBIs are one of the leading causes of death within this population and must be identified promptly to achieve optimal outcomes.^3^–^7^

Cranial computed tomography (CT) scanning is highly sensitive for identifying brain injuries.^8^ CT scanning is used with increasing regularity in the pediatric population to exclude intracranial brain injuries, with up to 69% of pediatric patients receiving a cranial CT scan.^9^,^10^ However, most cranial CT scans in blunt trauma patients are normal, less than 8% reveal intracranial brain injury, and even fewer require acute intervention.^11^,^12^ Moreover, overuse of CT scanning in children is concerning due to the risk of radiation exposure.^13^–^15^

In 2001, the American College of Radiology noted that because children have longer life expectancies and their cells divide more rapidly, they have higher radiation sensitivity which can lead to a greater risk of later malignancy than occurs in adults.^16^ In addition, growing evidence indicates that children undergo cranial CT scans when it may not always be necessary.^9^–^15^ In response, the Joint Commission issued a Sentinel Event Alert, reminding practitioners to adhere to the ALARA (as low as reasonably achievable) guidelines mandated by the Nuclear Regulatory Commission.^14^

Currently, there is a lack of consensus regarding which pediatric MTBI patients require a CT scan, especially among younger children who present with a minor head injury or concussion (Glasgow Coma Scale [GCS] score of 13 – 15). An example of this lack of consensus is apparent when examining the utilization of pediatric head CT scans within general emergency departments (22%) when compared to pediatric emergency departments (13%).^9^ In addition, multiple studies of pediatric head trauma patients vary considerably on which clinical indicators are best at predicting which children are at low-risk for a traumatic brain injury, thus do not require a head CT. 11,17–26

Clinical indicators that have appeared in these studies include mechanism severity, physical examination findings of a basilar skull fracture, non-frontal scalp hematoma, low GCS score, loss of consciousness (LOC), neurologic deficits, altered mental status, prolonged vomiting, severe headache, amnesia, irritability, behavioral changes, seizures, and lethargy.^11^,^17^–^26^ The purpose of this study was to determine if the clinical indicators identified in previous studies were present among pediatric patients with a concussion and who had received a head CT scan.

## Patients and Methods

A retrospective review was conducted of patients younger than or equal to four years presenting to a single Midwestern American College of Surgeons-verified Level 1 Trauma Center between January 1, 2004 and December 31, 2010 following concussion due to blunt head trauma. Patients who died within the first 24 hours of admission, arrived intubated (since head CT would be indicated in this population regardless), and did not have a traumatic brain injury (isolated or non-isolated concussion) were excluded.

Demographic data collected included age and gender. Other collected data included: Injury Severity Score (ISS), head and neck Abbreviated Injury Score (AIS), GCS score, individual injury details, cranial CT scan results, and neurological surgeries performed in the hospital. Clinical indicators assessed in this study included: mechanism of injury (severe or not severe as defined below), physical examination findings of basilar skull fracture (raccoon eyes, Battle’s sign, hemotympanum, cerebrospinal fluid from ear/nose), non-frontal scalp hematoma, GCS score less than 15, loss of consciousness, presence of neurological deficit, altered mental status, prolonged vomiting, severe headache, amnesia, irritability, behavioral changes, seizures, and lethargy. Assessed outcomes included: intensive care unit (ICU) admission, ICU length of stay, mechanical ventilator days, need for re-intubation, hospital length of stay, in-hospital mortality, discharge destination, and need for re-admittance to hospital.

Severe mechanism of injury was defined as a motor vehicle collision (MVC) at 40 mph or greater or when the speed was unknown, and when there was an ejection, rollover, or death. Patients struck by a high-impact object or by a motorized vehicle, while either on foot or on a bicycle, also were included. Type of falls included were those of more than three feet for patients younger than two years, and more than five feet for patients older than two years. Falls of unknown height, from more than five stairs or unknown amount of stairs, and falls from a bicycle without a helmet also were included. Finally, patients suspected of being the victim of child abuse were included.

## Statistical Analysis

Descriptive analyses were presented as frequencies with percentages for categorical variables and means with standard deviations for continuous variables. Primary comparisons were made between patients with negative CT findings versus those with positive CT findings. Continuous variables were compared using one-way analysis of variance for normally distributed data. When heterogeneity of variance was identified, the Mann-Whitney U Test was utilized for analyses. Categorical data were compared using Chi-square analysis or the Fisher’s exact test when sample size was small. All tests were two-tailed, and a p < 0.05 was considered statistically significant. All statistical analyses were conducted using SPSS software, version 19.0 (IBM Corp., Somers, New York). This study was approved for implementation by the Institutional Review Board of Via Christi Hospitals Wichita, Inc. and the Human Subjects Committee at the University of Kansas School of Medicine-Wichita.

## Results

Initially, 189 patients were identified from the trauma registry. A total of 19 patients were excluded from data analyses. Nine were excluded from the study due to being older than four years or having a mechanism other than blunt head trauma. Another nine were excluded because they arrived intubated. One child was excluded due to having a chronic head bleed from an arteriovenous malformation found on imaging studies.

Of the remaining 170 children, most were male (62.9%, n = 107) with a mean age of 28.1 ± 15.9 months (range 0 to 59 months). Most patients presented to the hospital with a median GCS of 15, ISS of 4, and head/neck AIS of 2. The majority of patients had a CT scan (78.2%, n = 133) of which 5.3% (n = 7) were positive for either a cranial fracture and/or bleeding. One patient had an initial head CT that was read as negative, with observation of hemotympanum; however, a follow-up CT demonstrated a resolving subdural hematoma. Clinical findings, CT results, and hospital course for patients with positive CT scans are presented in Table 1.

A comparison of demographics, injury severity, and mechanism of injury between the two groups is shown in Table 2. Demographics, GCS, and ISS were similar between the study groups. Head/neck AIS was greater in the positive CT group (2.7 ± 0.9 vs. 1.9 ± 0.5, respectively, p = 0.002). Most patients (63.2%) were injured as a result of a fall. However, there was no difference between the study groups in regards to mechanism of injury.

Hospital outcomes for the study groups are compared in Table 3. Almost all of the patients with positive CT findings were admitted to a pediatric ICU (85.7%, n = 6), a higher proportion than among patients with negative CT findings (38.1%, n = 48, p = 0.018). There was no difference between the groups for intensive care unit length of stay and hospital length of stay. No neurosurgical procedures and no deaths occurred among the study population. All seven of the children with positive CTs and 97.6% (n = 123) of those with a negative CT were discharged home after treatment.

A comparison of the prevalence of clinical indicators between the study groups is shown in Table 4. Most patients had at least one clinical indicator present (95.4%, n=127). Of the clinical indicators studied, severe mechanism was the most common among the total patient population that received a CT scan (57.1%, n = 76), followed by loss of consciousness (38.3%, n = 51), GCS less than 15 (31.6%, n = 42), and lethargy (26.3%, n = 35). Among the positive CT group, each patient had at least one clinical indicator present on arrival, with six patients having two or more clinical indicators present.

Clinical indicators that were observed more commonly in patients with positive CT findings than in those with negative CT findings included severe mechanism (100% vs. 54.8%, respectively, p = 0.020) and signs of a basilar skull fracture (28.6% vs. 0.8%, respectively, p = 0.007). No other clinical indicators were significantly different between the two groups. Severe mechanism alone was found to be sensitive, but not specific, whereas signs of a basilar skull fracture, headache, behavioral changes, and vomiting were specific, but not sensitive (Table 5).

A subcategory of children with a minor TBI (GCS = 13 – 15) represented 94.7% of the total population (n = 161). The remaining 5.3% (n = 9) had a GCS less than 13 and were considered to have either moderate or severe TBI. Among those with a minor TBI, 77% (124/161) had a head CT performed. Seven of these head CT scans were positive for fractures and/or bleeds.

## Discussion

Literature supports the use of clinical indicators for screening children to determine when to perform a head CT scan.^11^,^17^–^26^ However, the clinical indicators that are most effective in determining the need for head CT scans in children remain controversial.^11^,^17^–^26^ In this retrospective study, more patients with a positive CT scan presented with a severe mechanism of injury and signs of basilar skull fracture than patients who had a negative CT scan. In addition, among the seven patients with positive head CT findings, at least one clinical indicator was present on arrival, with six of the seven patients having two clinical indicators present on arrival. Having more than one clinical indicator increases the risk of TBIs substantially.^17^,^18^

In the current study, signs of basilar skull fracture had the highest predictive value when compared to the other clinical indicators. This is in agreement with previous studies which have demonstrated an association between skull fractures in children and an increased risk of intracranial injuries.^11^,^17^–^26^ Alhelail et al.^19^ demonstrated that signs of basilar skull fractures were associated positively with the presence of subarachnoid hemorrhage, herniation, and cerebral edema. In the present study, among the two patients with a positive CT scan and signs of a basilar skull fracture, one patient had a fracture on their initial scan. The second patient had an original finding of hemotympanum with a subsequent finding of a subdural hematoma.

In addition, the current study results demonstrated that all patients with positive head CT scans suffered a severe mechanism of injury, making it the most common clinical indicator present. Consistent with previous studies, this study also found severe mechanism of injury as a common indicator of TBIs.^18^,^20^,^21^ However, most of these studies indicated that a combination of clinical indicators is needed to predict a TBI. For example, Nigrovic et al.^17^ concluded that children with an isolated severe mechanism of injury had a lower rate of clinically important TBIs than those with a severe mechanism of injury plus an additional clinical indicator. In our study, severe mechanism of injury was not specific for sustaining an intracranial injury as the majority of children with severe mechanisms had normal head CT findings.

Two clinical indicators that were not encountered among this study population included amnesia and neurological deficit. The absence of findings pertaining to amnesia may be due to the fact that the patients or patients’ families had not been asked specifically about the condition. More likely, amnesia may be a difficult finding to establish in the younger pediatric population. Alternatively, the absence of patients with neurological deficits may be due to the study’s focus on blunt head trauma, as well as the exclusion of patients who arrived intubated.

Most of the pediatric patients in the current study had a head CT (78.2%), with 5.3% of these scans being positive. The CT rate in other studies ranges from a low of 20%, up to 98%.^11^,^17^–^26^ However, the majority of our population also had at least one clinical indicator present regardless of CT results. A better judgment of our CT rate, based on using clinical indicators as a guide, is to look at the six patients who did not have a clinical indicator present. Reasons for why these patients may have received a CT despite not having any clinical indicators may include patient age, other clinical findings, physician discretion, or a request from the consulting physician and/or a parent.^27^ Due to the retrospective nature of this study, however, this information was not collected.

Among the twelve documented clinical indicators in our study, severe mechanism of injury and signs of basilar skull fracture were the only significantly different clinical indicators between the two populations, despite most of the total population demonstrating at least one indicator. In addition, all the clinical indicators that were present in the positive CT group were also present in the negative CT group. There were also several clinical indicators (seizures, altered mental status, irritability, lethargy and non-frontal scalp hematoma) that were only documented in the negative CT group.

These findings may indicate a need for change in diagnostic management among the youngest patients with MTBI. Among patients with clinical indicators, the risk of radiation exposure from a head CT may be warranted due to the risk of skull fracture or bleed. However, based on our findings, children without positive CT findings presented with clinical indicators. Other methods may need to be in place to limit radiation exposure. For instance, Atabaki et al.^18^ noted that some predictors in isolation (severe mechanism of injury, loss of consciousness, vomiting, headache) have a lower risk for clinically important traumatic brain injuries and advocate observation before CT use in these cases. In addition, CT is standard protocol in child abuse cases for ages two and under and application of these indicators would not decrease head CT use in this series. In the current study, four known child abuse cases were identified.

One unique patient in the study had an initial negative head CT with observation of hemotympanum, and a follow-up CT that demonstrated a resolving subdural hematoma. However, this patient had fluid in the basilar air cells on the initial head CT, which should be considered as indirect evidence for a basilar skull fracture. This was the only patient in the study who demonstrated a false-negative finding based upon initial head CT scan. Regardless, this patient demonstrated two clinical indicators for head CT scan (severe mechanism and signs of basilar skull fracture), and the finding of hemotympanum on initial head CT scan would have prompted physicians to perform a repeat head CT scan for diagnosis.

There were several limitations to this study, foremost was its relatively small sample size. Second, the lack of follow-up information available after patients were discharged precluded knowledge of long-term outcomes following dismissal. Third, data regarding patients who did not undergo a cranial CT scan were not reported, therefore, an assumption was made that these patients were without significant cranial injury. Finally, since this was a retrospective chart review, there were known limitations of documentation. One example was the difficulty in obtaining a length of time for those patients experiencing a greater than five-second period of loss of consciousness. Although, loss of consciousness was found to be a frequent clinical indicator for head CT scan, the duration rarely was documented within the medical record, making it a difficult clinical indicator to use in the context of a retrospective study.

## Conclusions

In the current study, most patients presented with at least one clinical indicator and most had a head CT scan. Severe mechanism of injury and signs of basilar skull fracture were more common for patients with a positive CT scan than patients with a negative CT scan. However, clinical indicators also were documented in patients with negative CT findings. This fact may indicate a need for change in diagnostic management among the youngest patients with MTBI.

## Figures and Tables

**Table 1:** Clinical findings, CT results, and hospital course of patients with positive CT Findings.

Patient	Clinical Findings	CT Results	Hospital Course
1	Ecchymosis right ear Severe mechanism Signs of basilar skull fracture Headache	Nondepressed left occipital calvarial fracture	Pediatric ICU length of stay: 1 day
2	Forehead bruise Severe mechanism Loss of consciousness	Nondisplaced, nondepressed linear skull fracture extending through the right occipital bone into the petrous ridge	Pediatric ICU length of stay: 1 day Neurosurgery consult
3	Forehead contusion Left forearm ecchymosis Severe mechanism Behavioral changes	Subarachnoid hemorrhage and parietal contusions	Pediatric ICU length of stay:1 day Neurosurgery consult Repeat CT: stable
4	Contusion scalp Severe mechanism Vomiting	Right parietal fracture extends into the temporal and petrous ridge and right mastoid	Pediatric ICU length of stay:1 day Neurosurgery consult Repeat CT: stable
5	Left frontal ecchymosis Severe mechanism Loss of consciousness Vomiting GCS 14	Tiny subdural hematoma	Pediatric ICU length of stay: 9 days Neurosurgery consult Skeletal series Bone scan MRI Repeat CT: stable
6	Severe mechanism Loss of consciousness	High parietal calvarial fracture extends from vertex down about 1.5 cm without intracerebral hemorrhage	Floor length of stay: 1 day Neurosurgery consult Repeat CT: stable
7	Open wound eardrum Severe mechanism Signs of basilar skull fracture	Hemotympanum	Pediatric ICU length of stay: 2 days ENT and neurosurgery consult Skeletal survey Repeat CT: small right subdural hematoma

**Table 2:** Comparison of patient demographics, injury severity, and mechanism of injury.

	Total CT	Positive CT	Negative CT	p value
Number of Observations	133 (100%)	7 (5.3%)	126 (94.7%)	---
Age (months)*	29.2 ± 16.2	23.1 ± 22.3	29.5 ± 15.9	0.316
Gender				0.710
Male	83 (62.4%)	5 (71.4%)	78 (61.9%)	
Female	50 (37.6%)	2 (28.6%)	48 (38.1%)	
Injury Severity				
Glasgow Coma Scale (GCS) Score*	14.6 ± 3.5	14.9 ± 0.4	14.6 ± 3.6	0.835
Injury Severity Score*	5.1 ± 3.9	8.4 ± 5.9	4.9 ± 3.7	0.328
Abbreviated Injury Severity Score head/neck*	1.9 ± 0.5	2.7 ± 0.9	1.9 ± 0.5	0.0002
Mechanism of Injury				0.192
Falls	84 (63.2%)	5 (71%)	79 (62.7%)	
Struck accidentally by object	15 (11.3%)	1 (14.3%)	14 (11.1%)	
Motor vehicle crash	27 (20.3%)	0	27 (20.3%)	
Suspected child abuse	5 (4.8%)	0	5 (4.0%)	
Pedal cycle accident	2 (1.5%)	1 (14.3%)	1 (0.8%)	

* Mean ± standard deviation

**Table 3:** Comparison of patient outcomes.

	Total CT	Positive CT	Negative CT	p value
Number of Observations	133 (100%)	7 (5.3%)	126 (94.7%)	
Hospital Course				
Intensive care unit (ICU) admission	54 (40.6%)	6 (85.7%)	48 (38.1%)	0.018
ICU length of stay, d*	1.3 ± 0.9	2.3 ± 3.3	1.2 ± 0.5	0.772
Hospital length of stay, d*	1.3 ± 0.9	2.3 ± 3.0	1.3 ± 0.8	0.305
Ventilator days*	---	---	1.0 ± 0.0	---
In-hospital deaths after 24 hours	0	0	0	---
Re-admissions	0	0	0	---
Procedures Performed				
Intubations	0	0	2 (1.6%)	1.000
Re-intubations	0	0	0	---
Neurosurgery	0	0	0	---
Discharged Destination				1.000
Home	130 (97.7%)	7 (100%)	123 (97.6%)	
Other (foster care, against medical advice)	3 (2.3%)	0	3 (2.4%)	

* Mean ± standard deviation

**Table 4:** *.* Comparison of patient clinical indicators.

	Total CT (n=133)	Positive CT (n=7)	Negative CT (n=126)	p value
Number Of observations*	127 (95.4%)	7 (100%)	120 (95.2%)	---
Severe Mechanism	76 (57.1%)	7 (100%)	69 (54.8%)	0.020
Loss of Consciousness	51 (38.3%)	3 (42.9%)	48 (40.3%)	1.000
GCS less than 15	42 (31.6%)	1 (14.3%)	41(32.5%)	0.312
Lethargy	35 (26.3%)	0	35 (27.8%)	0.189
Vomiting	26 (19.5%)	2 (28.6%)	24 (19.0%)	0.622
Behavioral Changes	10 (7.5%)	1 (14.3%)	9 (7.1%)	0.429
Seizures	10 (7.5%)	0	10 (7.9%)	1.000
Altered Mental Status	9 (6.8%)	0	9 (7.1%)	1.000
Irritability	8 (6.0%)	0	8 (6.3%)	1.000
Headache	8 (6.0%)	1 (14.3%)	7 (5.6%)	0.359
Signs of Basilar Skull Fracture	3 (2.3%)	2 (28.6%)	1 (0.8%)	0.007
Non-frontal Hematoma	3 (2.3%)	0	3 (2.4%)	1.000
Neurological Deficit	0	0	0	---
Amnesia	0	0	0	---

* A patient may have more than one indicator present.

**Table 5:** Sensitivity and specificity of clinical indicators based upon initial positive CT findings.

	Number	Sensitivity	Specificity	Positive predicative value	Negative predicative value
Clinical Indicators	7	1.00	0.05	0.06	1.00
Severe Mechanism	7	1.00	0.45	0.09	1.00
Loss of Consciousness	3	0.43	0.62	0.06	0.95
Vomiting	2	0.29	0.81	0.08	0.95
Signs of Basilar Skull Fracture	2	0.29	0.99	0.67	0.96
GCS < 15	1	0.14	0.67	0.02	0.93
Headache	1	0.14	0.94	0.13	0.95
Behavioral Changes	1	0.14	0.93	0.10	0.95
